# Celecoxib suppresses hepatoma stemness and progression by up-regulating PTEN

**DOI:** 10.18632/oncotarget.1745

**Published:** 2013-12-28

**Authors:** Tian-Huei Chu, Hoi-Hung Chan, Hsiao-Mei Kuo, Li-Fen Liu, Tsung-Hui Hu, Cheuk-Kwan Sun, Mei-Lang Kung, Shih-Wei Lin, E-Ming Wang, Yi-Ling Ma, Kwan-Hung Cheng, Kwok Hung Lai, Zhi-Hong Wen, Ping-I Hsu, Ming-Hong Tai

**Affiliations:** ^1^ Institute of Biomedical Sciences, National Sun Yat-sen University, Kaohsiung, Taiwan; ^2^ Department of Biological Sciences, National Sun Yat-sen University, Kaohsiung, Taiwan; ^3^ Division of Gastroenterology, Department of Internal Medicine, Kaohsiung Veterans General Hospital, Kaohsiung, Taiwan; ^4^ School of Medicine, National Yang-Ming University, Taipei, Taiwan; ^5^ College of Pharmacy & Health Care, Tajen University, Pingtung County, Taiwan; ^6^ Mitochondrial Research Unit, Kaohsiung Chang Gung Memorial Hospital and Chang Gung University College of Medicine, Kaohsiung, Taiwan; ^7^ Department of Biological Science and Technology, I-Shou University, Kaohsiung, Taiwan; ^8^ Division of Hepato-Gastroenterology, Department of Internal Medicine, Chang Gung Memorial Hospital Kaohsiung Medical Center, Chang Gung University College of Medicine, Kaohsiung, Taiwan; ^9^ Department of Medical Education, E-DA Hospital, I-Shou University, Kaohsiung, Taiwan; ^10^ Department of Chemistry, National Sun Yat-sen University, Kaohsiung, Taiwan; ^11^ Institute of Marine Biotechnology, National Sun Yat-sen University, Kaohsiung, Taiwan; ^12^ Department of Medical Education; Digestive Center, E-DA Hospital, Kaohsiung County, Taiwan; ^13^ Department of Marine Biotechnology and Resources, Asia-Pacific Ocean Research Center, National Sun Yat-Sen University, Kaohsiung, Taiwan; ^14^ Center for Neuroscience, National Sun Yat-Sen University, Kaohsiung 804, Taiwan

**Keywords:** hepatocellular carcinoma, hepatic cancer stem cells, celecoxib, prostaglandin E2, phosphatase and tensin homolog

## Abstract

Celecoxib, a COX-2 inhibitor and non-steroidal anti-inflammatory drug, can prevent several types of cancer, including hepatocellular carcinoma (HCC). Here we show that celecoxib suppressed the self-renewal and drug-pumping functions in HCC cells. Besides, celecoxib depleted CD44 + /CD133 + hepatic cancer stem cells (hCSC). Prostaglandin E2 (PGE2) and CD133 overexpression did not reverse the celecoxib-induced depletion of hCSC. Also, celecoxib inhibited progression of rat Novikoff hepatoma. Moreover, a 60-day celecoxib program increased the survival rate of rats with hepatoma. Histological analysis revealed that celecoxib therapy reduced the abundance of CD44 + /CD133 + hCSCs in hepatoma tissues. Besides, the hCSCs depletion was associated with elevated apoptosis and blunted proliferation and angiogenesis in hepatoma. Celecoxib therapy activated peroxisome proliferator-activated receptor γ (PPARγ) and up-regulated *PTEN*, thereby inhibiting Akt and disrupting hCSC expansion. PTEN gene delivery by adenovirus reduced CD44/CD133 expression *in vitro* and hepatoma formation *in vivo*. This study suggests that celecoxib suppresses cancer stemness and progression of HCC via activation of PPARγ/PTEN signaling.

## INTRODUCTION

Hepatocellular carcinoma (HCC) accounts for 70–85% of liver cancers and is one of the most common malignancies worldwide [1]. Current HCC therapies include surgery, liver transplantation, chemotherapy, transarterial chemoembolization (TAE), and radiofrequency ablation [2]. However, the overall prognosis for HCC remains poor. Sorafenib, an inhibitor of multiple kinases, including vascular endothelial growth factor receptor (VEGFR), platelet-derived growth factor receptor (PDGFR), and Raf kinases, is currently the only target therapy for HCC [3, 4]. However, the high cost and limited pro-survival effect of sorafenib warrant further development of therapeutic alternatives for HCC.

Cyclooxygenase-2 (COX-2) is an inducible enzyme frequently found in inflammatory tissues and is involved in carcinogenesis pathways in many organs. It has been reported that COX-2 expression is correlated with angiogenesis, invasion, relapse, chemoresistance, and tumorigenesis in HCC [5]. Besides, a significant correlation between COX-2 expression and active inflammation in the adjacent non-cancerous liver is associated with shorter disease-free survival in HCC patients [6]. COX-2 promotes hepatoma cells growth and inhibits cell apoptosis through Akt activation [7, 8]. Prostaglandin E2 (PGE_2_), the major product of COX-2, stimulates the proliferation, migration, and invasion in hepatoma cells by activating β-catenin and Akt signaling [9].

Cancer stem cells (CSCs), the rare and most malignant subpopulations in tumors, are maintained by indefinitely self-renew abilities and resistant to drug therapy [10, 11]. CD133 is a universal marker of stem cells and CSCs and CD133^+^ hepatic cancer stem cells (hCSCs) tumor cells have been identified to exhibit properties of CSCs in human HCC and mouse liver cancer [12-14]. Indeed, CD133^+^ hCSCs cells have higher expression and increased activity of aldehyde dehydrogenase (ALDH) to promote their tumorigenicity [13]. Moreover, a recent study has indicated that high expression levels of hCSCs biomarkers, including CD133 and CD44, are correlated with tumor angiogenesis and poor prognosis of HCC patients [15]. Though not fully elucidated, several signaling pathways, including Akt and Wnt/β-catenin, have been implicated in the CSCs maintenance [11]. Besides, inhibition of Akt signaling leads to decreased CD133 and CD44 expression in HCC cells [16, 17].

Celecoxib, a selective COX-2 inhibitor and non-steroidal anti-inflammatory drug (NSAID), is widely used for pain and inflammation. Celecoxib attenuates Akt phosphorylation and induces growth inhibition and apoptosis in HCC cells, which can be partially reversed by ectopic COX-2 expression and PGE_2_ [8]. The chemopreventive effect of celecoxib has been demonstrated in animal models of diethylnitrosamine-induced HCC [18] and Huh7 xenograft hepatoma [19]. Both COX-2-dependent and COX-2-independent pathways contribute to celecoxib-mediated HCC chemoprevention [20]. However, the therapeutic efficacy of celecoxib for HCC has not been tested in immune-competent animals with orthotopic hepatoma. Thus, this study investigated the therapeutic potential and mechanism of celecoxib in rat Novikoff hepatoma induced by US-guided implantation [21].

## RESULTS

### PGE2 from non-tumor tissues induces differential distribution of liver cancer stem cells in Novikoff hepatoma

**Fig 1:**
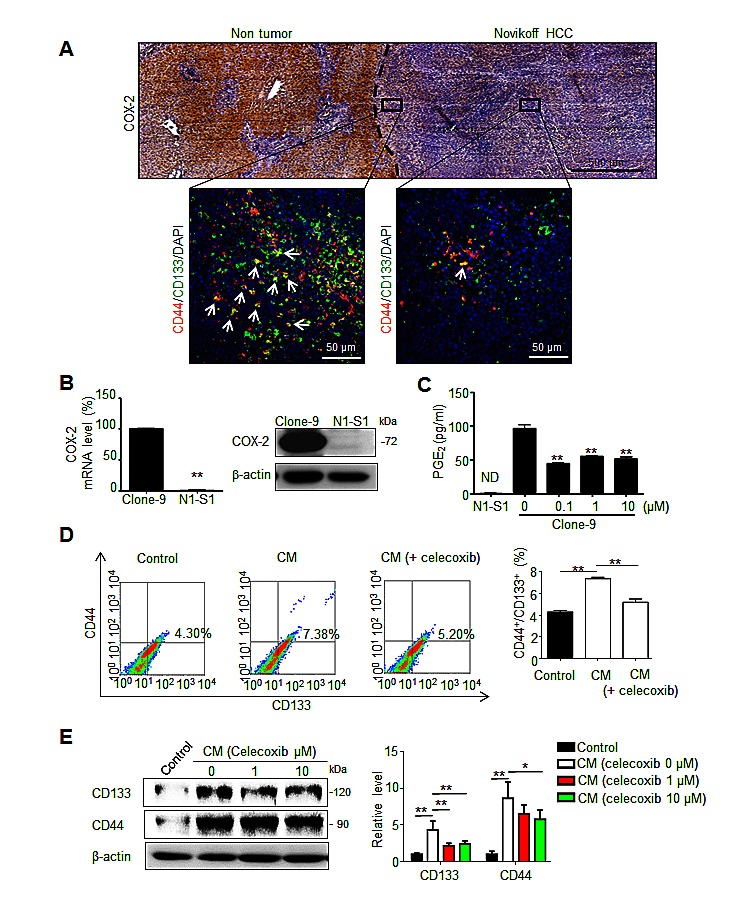
PGE2 from non-tumor tissues enhanced the cancer stemness of Novikoff HCC (A) Histological analysis of COX-2 expression and CD44^+^/CD133^+^ hCSCs distribution in Novikoff hepatoma. (Top panel) Immunohistochemical studies showed elevated COX-2 expression in non-tumor hepatic tissues compared with that in Novikoff hepatoma. (Bottom insets) Immunofluorescence analysis revealed higher abundance of CD44^+^/CD133^+^ hCSCs in regions adjacent to non-tumor tissues (left inset) than the farther away ones (right inset). White arrow indicated CD44^+^/CD133^+^ hCSCs. (B) Quantitative RT-PCR and immunoblot analysis of COX-2 expression in Clone-9 and N1-S1 cells. (C) EIA analysis of PGE_2_ secretion in N1-S1 cells and Clone-9 cells after treatment with varying doses of celecoxib for 48 h. ND, not detectable. (D) Conditioned media (CM) of Clone-9 cells were exposed to 10 μM celecoxib for 48 h in 0.1% serum F-12K medium, and then N1-S1 cells were incubated in Clone-9 conditioned medium (CM) for an additional 48 h. Next, 0.1% serum F-12K medium was incubated for 48 h to prepare control medium for N1-S1 co-culture. After N1-S1 cells were cultured in celecoxib-treated Clone-9 culture medium (CM) or control medium, N1-S1 cells were collected to evaluate the ratio of CD44^+^/CD133^+^ cells using flow cytometry, and statistic results are shown. (E) Immunoblot analysis for CD44 and CD133 in N1-S1 cells treated with control medium or CM, and statistic results are shown. Data are mean ± SD (*p < 0.05, **p < 0.01).

Poorly differentiated HCCs expressed less COX-2 than surrounding hepatocytes of non-tumor region [22], and COX-2 expression in non-tumor tissue play a positive role in relapse of HCC after surgery [6]. We first examined COX-2 expression and the spatial distribution of CD44^+^/CD133^+^ hCSCs in Novikoff hepatoma using immunohistochemical and immunofluorescent analyses, respectively. COX-2 immunostaining was primarily localized in non-tumor hepatic tissues, but was only minimally detected in Novikoff hepatoma. Moreover, CD44^+^/CD133^+^ hCSC abundance was increased in regions near COX-2-expressing non-tumor tissues compared with regions that were farther away (Fig. 1A). Based on the lack of COX-2 expression in hepatoma, it was hypothesized that PGE_2_ originated from the COX-2-expressing liver and promoted the generation of cancer stem cells in Novikoff hepatoma. To test this hypothesis, comparative analysis of COX-2 expression between N1-S1 rat hepatoma cells and Clone-9 rat liver cells showed that COX-2 mRNA and protein levels were significantly higher in Clone-9 cells than in N1-S1 cells (Fig. 1B). Additionally, COX-2-expressing Clone-9 cells secreted significantly higher amounts of PGE_2_, whose production was significantly inhibited by treatment with celecoxib (Fig. 1C). PGE_2_ secretion was not detectable in N1-S1. We then investigated whether conditioned medium (CM) from Clone-9 cells, which contained a high PGE_2_ content, influenced the generation of hCSCs in N1-S1 cells. Flow cytometry analysis showed that co-culture with CM from Clone-9 cells significantly enhanced the abundance of CD44^+^/CD133^+^ hCSCs in N1-S1 cells (Fig. 1D). This stimulatory effect was diminished in CM from celecoxib-treated Clone-9 cells, indicating that PGE_2_ has a stimulatory function in hCSC genesis. Moreover, immunoblot analysis showed that incubation with CM from Clone-9 cells elevated expression of CD44 and CD133 in N1-S1 cells (Fig. 1E), whereas CM from celecoxib-treated clone-9 cells upregulated CD44 and CD133 to a lesser extent. These results suggest that PGE_2_ from hepatic tissues induces differential hCSC distribution in Novikoff hepatoma.

### Celecoxib suppresses the function and abundance of cancer stem-like cells in HCC cells

**Fig 2:**
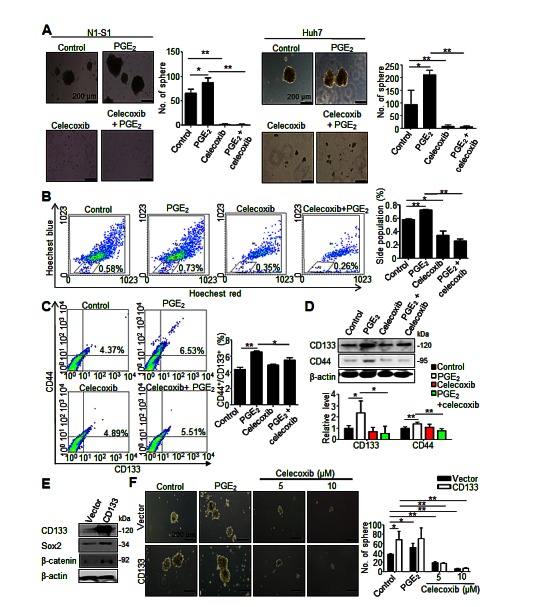
Celecoxib inhibited the function and abundance of hCSC in HCC cells (A) Effect of celecoxib on spheres formation in N1-S1 and Huh7 cells with or without PGE_2_ pretreatment. (B) Effect of celecoxib on side population cells in N1-S1 cells with or without PGE_2_. (C) Flow cytometry analysis of the effect of celecoxib on CD133^+^/CD44^+^ hCSCs in N1-S1 cells with or without PGE_2_. (D) Immunoblot analysis of the effect of celecoxib on CD133 and CD44 expression in N1-S1 cells with or without PGE_2_. (E) Immunoblot and (F) sphere formaiton analysis in retro-CD133 or retro-vector infectd-N1-S1 cells. Data were mean ± SD (*p < 0.05, **p < 0.01).

We first evaluated the anti-neoplastic efficacy of celecoxib *in vitro* by cell proliferation assay and colony formation assay, respectively. Despite moderate inhibition on cell proliferation (Supplementary Fig. 1A and B), celecoxib inhibited the oncogenic behaviors, including invasiveness (data not shown) and anchorage-independent growth (Supplementary Fig. 1C and D), in human Hep3B and Huh7 cells respectively with a half-maximal inhibitory concentrations (IC_50_) of 31.32 and 29.33 μM. We subsequently investigated the influence of celecoxib on hCSCs functions in HCC cells in the absence or presence of PGE_2_. By using sphere formation assay to evaluate the self-renewal capability of hCSCs [23], it was found that celecoxib potently inhibited the basal or PGE_2_-stimulated sphere formation in human (Huh-7) and rat (N1-S1) HCC cells (Fig. 2A). To study the drug efflux functions of hCSCs using side population (SP) analysis [24], it was observed celecoxib also attenuated the endogenous and PGE_2_-induced drug-pumping capability in N1-S1 cells (Fig. 2B). Together, these results indicated that celecoxib perturbs the hCSCs functions in HCC cells.

We then evaluated whether celecoxib affected the abundance of CD44^+^/CD133^+^ hCSCs in hepatoma cells. PGE_2_ is a potent stem cells regulator, and it can promote stem cells function [25]. We also found that exogenous PGE_2_ induced up-regulation of CD44 and CD133 hCSCs marker in human Hep3B and Huh7 cells (Supplementary Fig. 2). Flow cytometry analysis showed that celecoxib significantly attenuated the PGE_2_-induced CD44^+^/CD133^+^ hCSCs in N1-S1 cells (Fig. 2C). Besides, celecoxib treatment significantly decreased the CD133 and CD44 protein levels in PGE_2_-stimulated N1-S1 cells (Fig. 2D). To test whether CD133 overexpression could revert the celecoxib-induced hCSCs depletion, we generated CD133-overexpressing N1-S1 cells using retrovirus gene delivery (Fig. 2E). These CD133-overexpressing N1-S1 cells exhibited elevated expression of CSC markers, including Sox2 and β-catenin, and enhanced self-renewal ability. Nevertheless, celecoxib supply still significantly abrogated the spheres formation of CD133-expressing N1-S1 cells at low concentration (5 μM; Fig. 2F). Moreover, combination of CD133 overexpression with excessive PGE_2_ failed to rescue the celecoxib-induced suppression of sphere formation in N1-S1 cells. Together, these results indicate that celecoxib is an effective blocker of cancer stem-like cells in HCC cells.

### Celecoxib therapy perturbs the tumor progression and prolongs the survival in rats bearing Novikoff hepatoma

**Fig 3:**
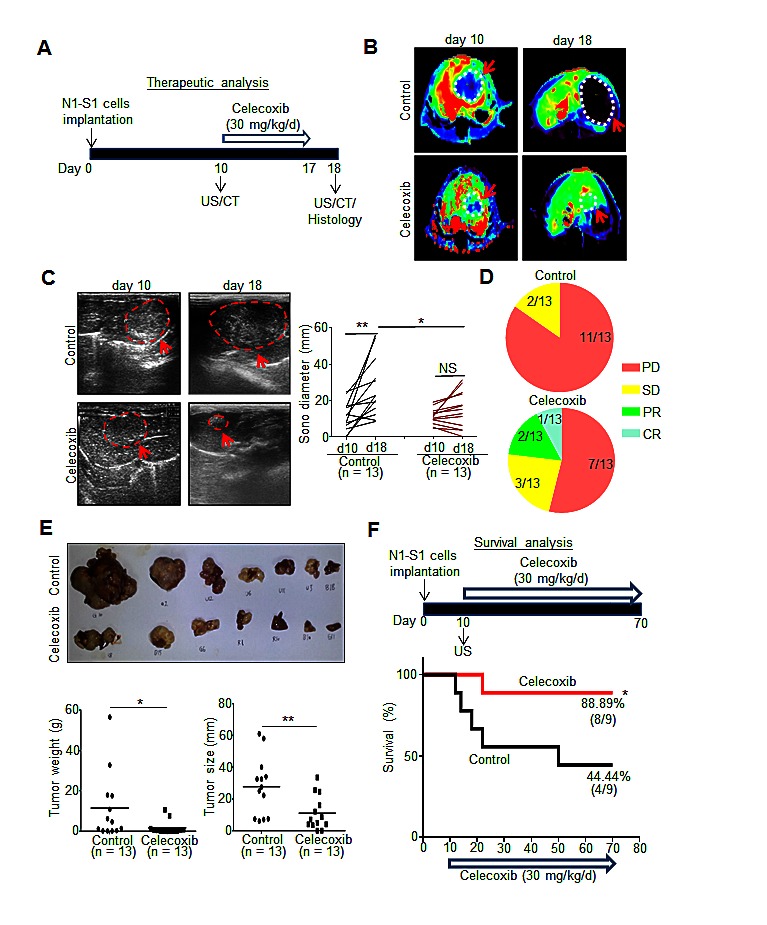
Therapeutic and survival effect of celecoxib in rats bearing established Novikoff hepatoma (A) Experimental scheme. (B) CT images analysis of rat Novikoff hepatoma before and after celecoxib therapy (arrows indicated the tumors; dotted line depicted the tumor areas). (C) US monitoring of rat Novikoff hepatoma before and after celecoxib therapy (left panel; arrows indicates the hepatoma; dotted line depicted the tumor areas). Statistical analysis of US-measured tumor sizes (right panel). (D) RECIST analysis for the response of celecoxib therapy. (PD, progressive disease; SD, stable disease; PR, partial response; CR, complete response). (E) Photographs of hepatic tumors, microbalance-measured tumor weight and caliper-measured tumor size after animal sacrificing. (F) Kaplan-Meier survival analysis. (*p < 0.05, **p < 0.01; NS, no significance).

Before testing the therapeutic efficacy, we evaluated the chemopreventive effect of celecoxib for Novikoff hepatoma in rats and found that a 17-day prophylactic celecoxib program significantly reduced the size and weight of Novikoff hepatoma compared with drinking water-treated control (Supplementary Fig. 3A-C). Subsequently, we investigated the therapeutic efficacy of a 7-day celecoxib regimen for established Novikoff hepatoma in rats by serial non-invasive CT and US analysis (Fig. 3A-C). Both CT and US imaging analysis revealed that celecoxib therapy perturbed hepatoma progression. Quantification analysis of US-measured hepatoma diameters showed that mean tumor size in celecoxib-treated group was not significantly increased (from 11.07 ± 4.30 mm on day 10 to 14.98 ± 10.16 mm on day 18; n = 13) whereas mean tumor size in control group was significantly increased within the same period (from 11.31 ± 8.03 to 26.55 16.02 mm; *p* < 0.01, n = 13). Moreover, the size of celecoxib-treated hepatoma was significantly smaller than that in control group (*p* < 0.05; Fig. 3C).

According to Response Evaluation Criteria in Solid Tumours (RECIST) ver.1.1 [26], patients with an increase of 20% or more in lesion size or those with new lesions were regarded as having progressive disease (PD). Patients with a change of lesion size ranging from an increase of <20% to a decrease of <30% and with no new lesion were stratified as having stable disease (SD). Patients with a 30% or greater decrease in the target lesion were regarded as achieving partial response (PR). Patients with disappearance of the lesion were stratified as achieving complete response (CR). RECIST analysis showed that 84.62% (11/13) animals showed PD, and 15.38% (2/13) animals showed SD in control group (Fig. 3D). In celecoxib-treated group, 53.85% (7/13) animals showed PD, 23.08% (3/13) animals showed SD, 15.38% (2/13) animals showed PR, and 7.69% (1/13) animals showed CR. Quantification studies of dissected hepatoma tissues also confirmed that the weight and size of hepatoma were significantly reduced in rats receiving celecoxib therapy (*p* < 0.05 and *p* < 0.01; Fig. 3E).

By using Kaplan-Meier analysis, the survival study indicated a 60-day celecoxib therapy significantly increased the survival rate in rats bearing established hepatoma (88.99% in celecoxib group versus 44.44% in control; *p* < 0.05; Fig. 3F). These findings supported the therapeutic potential of celecoxib in rats with pre-existing hepatoma.

### Celecoxib therapy disrupted the hCSCs markers expression and genesis in Novikoff hepatoma

**Fig 4:**
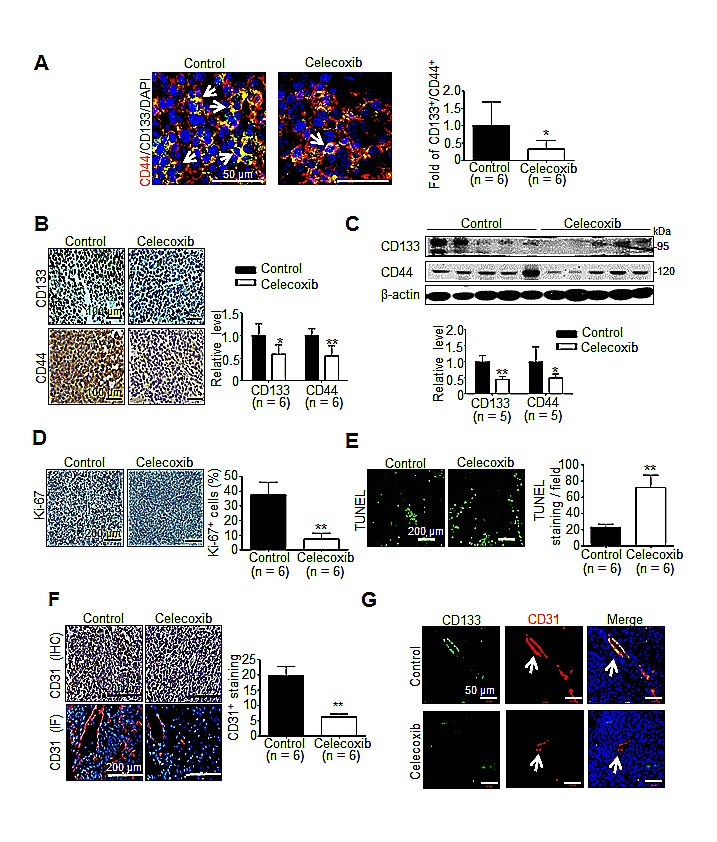
Effect of celecoxib therapy on cancer stemness, proliferation, apoptosis and angiogenesis in Novikoff hepatoma (A) Immunofluorescence staining (white arrow indicates CD44^+^/CD133^+^ hCSCs in tumor tissues), (B) Immunohistochemestry staining (left panel) and (C) immunoblots (right panel) for CD133 and CD44 from tumor tissues after celecoxib therapy for 7 days. Immunohistological studies of (D) Ki-67, (E) TUNEL and (F) CD31 in control or celecoxib-treated liver tumor. (G) Immunofluorescence analysis for CD133^+^/CD31^+^ endothelium cells in hepatic tumors (white arrow indicates blood vessels in the tumor tissues). (*p < 0.05, **p < 0.01).

To investigate whether the anti-tumor effect was associated with the depleted cancer stem cells in celecoxib-treated hepatoma, immunofluorescence analysis reveale that the significantly decreased prevalence of CD133^+^/CD44^+^ hCSCs in celecoxib-treated hepatoma than control group (Fig. 4A). Immunohistochemical analysis showed that the immunostaining of CD133 and CD44 was significantly reduced in celecoxib-treated hepatoma compared with that in control (Fig. 4B). This was consistent with the results of immunoblot analysis, which showed a significantly decreased CD133 and CD44 protein levels in celecoxib-treated hepatoma tissues (Fig. 4C). Interestingly, prophylactic celecoxib regimen also suppressed the expression of CD133 and CD44 in tumor tissues (Supplementary Fig. 3D and E). Therefore, celecoxib therapy attenuated the expression of hCSC markers, thereby depleting the hCSC genesis in Novikoff hepatoma.

### Celecoxib therapy elicits growth inhibition, apoptosis and angiogenesis blockade in Novikoff hepatoma

Histological analysis was performed to delineate the anti-neoplastic mechanism of celecoxib therapy other than perturbing cancer stemness. By immunostaining of Ki-67, an index of cell proliferation, it was observed that celecoxib therapy significantly decreased the Ki-67-positive proliferating cells in hepatoma tissues (Fig. 4D). This was accompanied with an increment in TUNEL-positive apoptotic cells (Fig. 4E). Since angiogenesis is critical to the viability of cancer cells, we evaluated the influence of celecoxib on neovascularization in hepatoma tissues. It was noted that the CD31-positive, neovascularized vessels were significantly reduced in celecoxi-treated Novikoff hepatoma (Fig. 4F). A recent study has indicated that CD133^+^ CSCs may give rise to tumor endothelium, thereby contributing to tumor vasculature and angiogenesis [27]. Interestingly, immunofluorescence staining unveiled that the CD133^+^/CD31^+^ vessels were significantly diminished in celecoxib-treated hepatoma (Fig. 4G).

### Celecoxib induces PPARγ/PTEN activation to disrupt Akt signaling and cancer stemness in hepatoma cells

**Fig 5:**
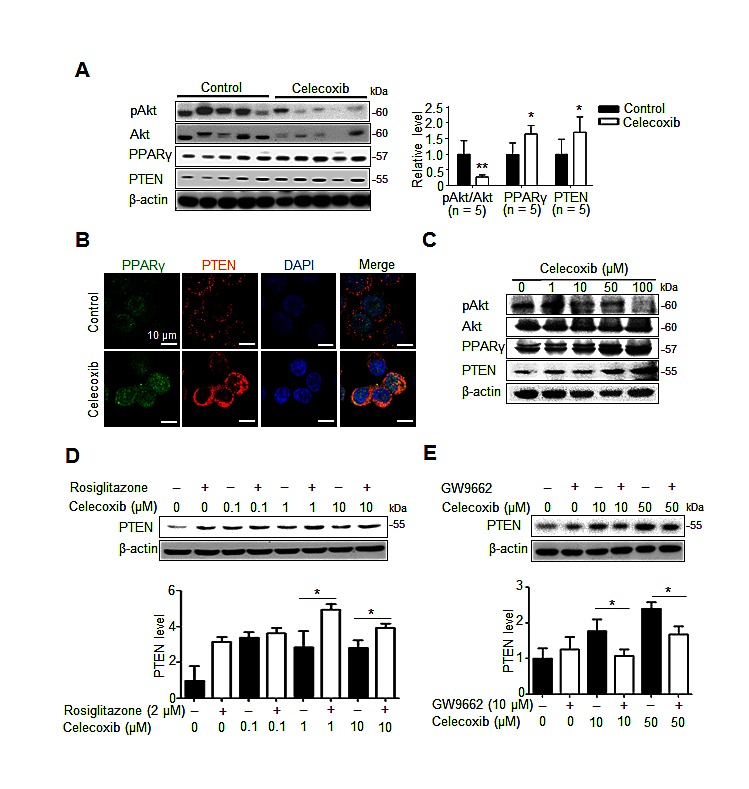
PPARγ/PTEN signaling contributes to celecoxib-induced Akt inhibition (A) Immunoblots from 7-day celecoxib-treated tumor tissues. (B) Immunofluorescence analysis in N1-S1 cells treated with 10 μM celecoxib for 48 h. (C) Immunoblots from N1-S1 cells treated with different dose celecoxib for 48 h. (D) Immunoblots from N1-S1 cells treated with celecoxib for 48 h in the presence or absence of 2 μM rosiglitazone. (E) Immunoblots from N1-S1 cells treated with celecoxib for 48 h in the presence or absence of 10 μM GW9662 (*p < 0.05, **p < 0.01).

Because Akt signaling is activated in CD133^+^ human HCC cells [16], we studied whether Akt pathway was involved in celecoxib-induced suppression of hepatic cancer stemness. Moreover, celecoxib has been reported to stimulate PPARγ as well as PTEN, the endogenous antagonist of Akt signaling, in hepatoma cells [20], we investigated whether this signaling pathway affects the therapeutic mechanism of celecoxib for Novikoff hepatoma. Immunoblot analysis revealed that expression of PPARγ and PTEN was significantly upregulated as well as Akt was dephoshorylated in celecoxib-treated hepatoma tissues (Fig. 5A). Likewise, immunofluorescence analysis revealed the prominent increase in PPARγ and PTEN expression in celecoxib-treated N1-S1 cells (Fig. 5B). Immunoblot analysis further showed that celecoxib-elicited PPARγ/PTEN upregulation and Akt dephosphorylation were dose-dependent (Fig. 5C).

Pharmaceutical modulators of PPARγ signaling were used to confirm the role of PPARγ in celecoxib-induced PTEN upregulation in hepatoma cells. Treatment with rosiglitazone, a PPARγ agonist, increased the endogenous and celecoxib-induced PTEN protein levels in hepatoma cells (Fig.5D). In contrast, adding GW9662, a PPARγ antagonist, attenuated celecoxib-induced PTEN upregulation without affecting the basal PTEN levels (Fig. 5E).

**Fig 6:**
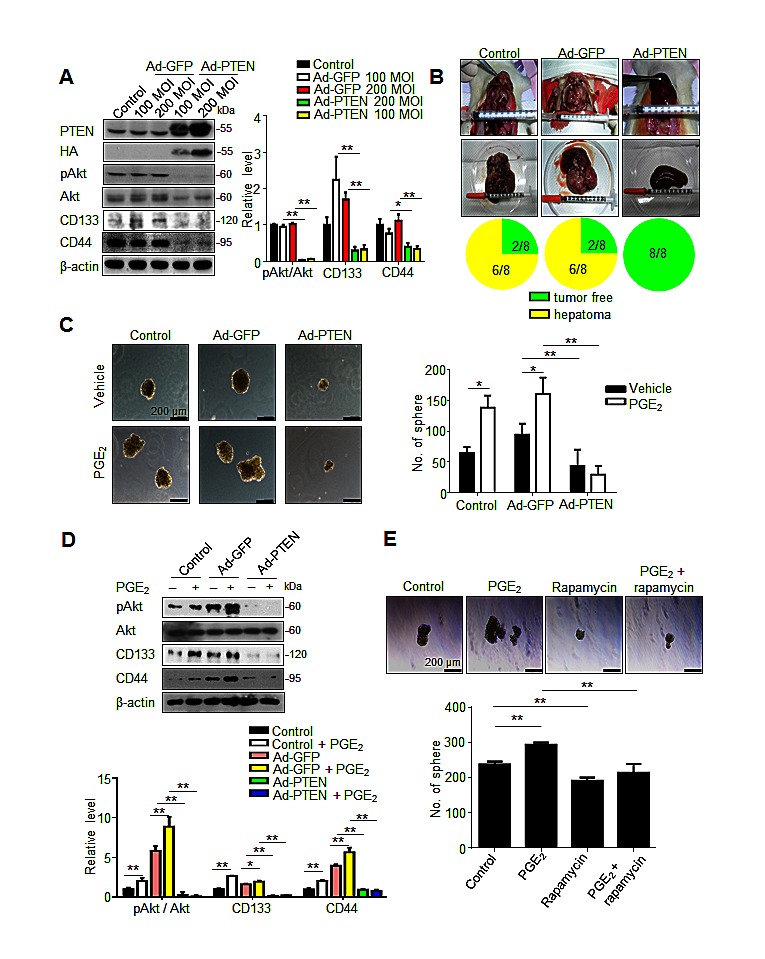
PTEN overexpression suppresses cancer stemness and tumor initiating (A) Immunoblot analysis in N1-S1 cells infected with Ad-GFP or Ad-PTEN for 48 h. (B) Effect of Ad-GFP (200 MOI) or Ad-PTEN (200 MOI) on tumor induction rate in orthotopic Novikoff hepatoma model. (C) Effect of gene delivery on spheres formation in Ad-GFP-infected (200 MOI) or Ad-PTEN-infected (200 MOI) Huh7 cells with or without PGE_2_ (1 μM) pretreatment. (D) Immunoblots from Ad-GFP- (200 MOI) or Ad-PTEN- (200 MOI) infected Huh7 cells treated with PGE_2_ (1 μM) for 48 h. (E) Effect of rapamycin (100 nM) on spheres formation in N1-S1 and Huh7 cells with or without PGE_2_ pretreatment. (*p < 0.05, **p < 0.01).

**Fig 7:**
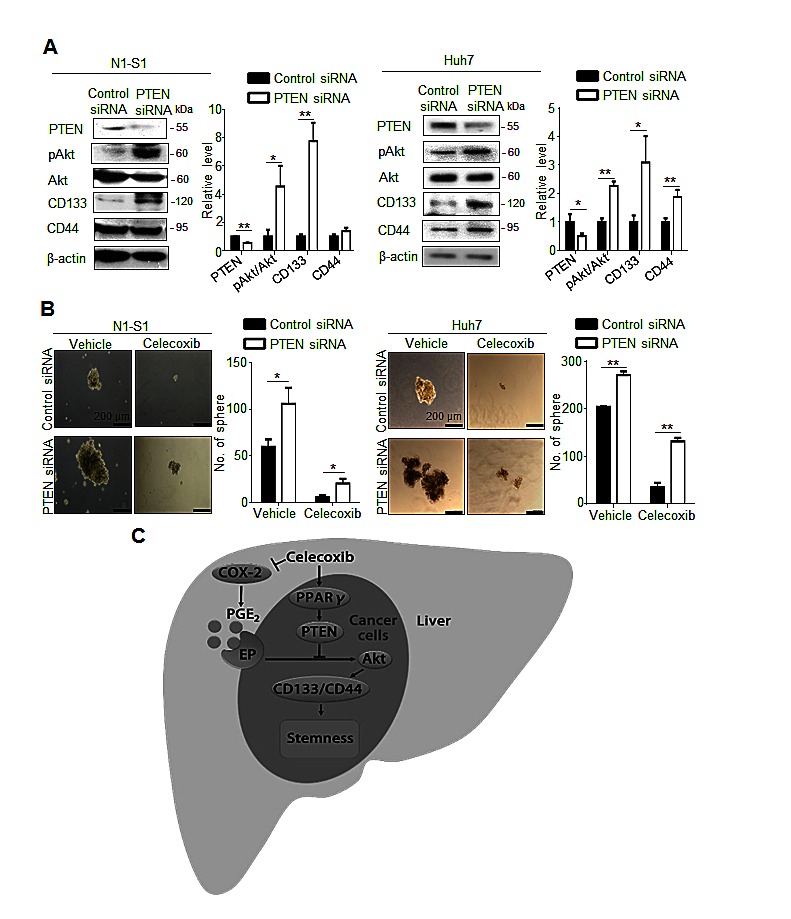
PTEN knockdown attenuates the effect of celecoxib on stemness (A) Immunoblot analysis in N1-S1 (left panel) and Huh7 (right panel) cells after siRNA transfection for 72 h. (B) Effect of celecoxib on spheres formation in control siRNA-trasfected- or PTEN siRNA-transfected- N1-S1 (left panel) and Huh7 cells (right panel). (C) The proposed mechanism for celecoxib-induced inhibition of hCSCs. (*p < 0.05, **p < 0.01).

To validate whether PTEN upregulation indeed contributed to the inhibition of hepatic cancer stemness and hepatoma growth, we employed adenovirus gene delivery to achieve PTEN overexpression in hepatoma cells. It was found that PTEN-overexpressing N1-S1 cells not only showed attenuated Akt phosphorylation, but also diminished CD133 and CD44 expression (Fig. 6A). In animal study, Ad-PTEN-infected N1-S1 cells did not led to hepatoma formation in rats (0/8), which whereas was lower than tumor incidence in Ad-GFP (6/8) and control (6/8) groups (Fig. 6B). Moreover, we also found that Ad-PTEN gene delivery suppressed PGE_2_-promoted sphere formation in human Huh-7 cells (Fig. 6C). In immunoblot analysis, PTEN overexpression also reversed PGE_2_-induced Akt activation and hCSCs marker up-regulation in Huh7 cells (Fig. 6D). Because mammalian target of rapamycin (mTOR) is downstream effector of Akt, we utilized rapamycin, a mTOR inhibitor, and found rapamycin also suppressed PGE_2_-stimulated sphere formation in hepatoma cells (Fig. 6E). To validate the role of PTEN in celecoxib-mediated stemness inhibition, PTEN knockdown was performed using PTEN siRNA, which significantly increased the expression of CD133 and CD44 (Fig. 7A), and enhanced the cancer stemness in hepatoma cells in HCC cells (Fig. 7B). Besides, PTEN silencing significantly attenuated the anti-stemness effect of celecoxib in N1-S1 cells (from 90.50% inhibition to 80.82% inhibition; *p* < 0.05) and Huh7 cells (from 82.84% inhibition to 51.41% inhibition; *p* < 0.01) cells. These results indicate PPARγ/PTEN/Akt signaling plays a crucial role in celecoxib-induced suppression of cancer stemness and hepatoma progression.

## DISCUSSION

The present study demonstrates for the first time that celecoxib is a potent inhibitor of cancer stemness in HCC *in vitro* and *in vivo*. This is consistent with a recent study that celecoxib inhibits tumor sphere through CD133 downregulation in colon cancer [28]. Importantly, the anti-CSCs function of celecoxib could not be reversed by exogenous PGE_2_ supply, implicating COX-2/PGE_2_-independent pathway might be involved in celecoxib-induced stemness suppression. This is in accordance with previous studies showing COX-2-dependent and COX-2-independent mechanism in celecoxib-mediated inhibition of HCC growth [19, 20]. However, celecoxib alone inhibited the stemness-related function without affecting the CD44^+^/CD133^+^ hCSCs, implicating additional hCSC population were regulated by celecoxib. We next validated whether PGE_2_-promoted and celecoxib-inhibited self-renewal were through the regulation of hCSCs surface markers in N1-S1 cells. CD133 over-expression significantly promoted the sphere formation, but the stimulatory effect of PGE_2_ on sphere formation could not be observed in CD133-overexpressing hepatoma cells, suggesting that CD133 up-regulation participated in PGE_2_-induced cancer stemness. However, CD133 overexpression failed to rescue celecoxib-induced suppression of sphere formation, implicating CD133 is might be a relatively downstream effector in celecoxib-induced stemness inhibition. Together, CD133 down-regulation contributed to celecoxib-induced suppression of PGE_2_-stimulated cancer stemness.

COX-2/PGE_2_ signaling regulates liver regeneration [29] and stem cells [25], and signal regulation and the pathway are similar for normal and cancer stem cells [30]. COX-2/PGE_2_ signaling can promote cancer stemness in colon cancer [31, 32], breast cancer [33] and leukemia [34]. COX-2 can promotes HCC cell growth through Akt activation [8], but the role of COX-2/PGE_2_ signaling in hepatic cancer stemness has not yet been reported. In this study, exogenous or non-tumor derived PGE_2_ promoted tumor sphere formation and increment of SPCs and CD44^+^/CD133^+^ hCSCs in HCC cells. It has been reported that PGE_2_ can promote Akt signaling [9], and Akt signaling also involved in CD133^+^ hCSCs regulaton [16]. From upon evidences, non-tumor COX-2 can be as a target for cancer therapy and prevention in advanced HCC.

Recently, lupeol, a triterpene from fruits and vegetables, inhibited the self-renewal ability of liver tumor-initiating cells (TICs) present in both HCC cell lines and clinical HCC samples. Besides, PTEN upregulation participates in lupeol-induced inhibition of CD133 expression, self-renewal and chemoresistance in hepatoma cells [23]. It also been reported that NSAIDs suppress cancer stem cells via inhibiting COX-2 and activating PPARγ in colon cancer [35]. Celecoxib also up-regulates PPARγ and PTEN, and PTEN is a negative regulator of Akt activity. It has been reported that celecoxib increases PPARγ expression and PTEN activity in wild-type and COX-2-deleted Huh7 cells [20]. Besides, two putative PPARγ binding sites within the PTEN promoter are present approximately 15 and 13 kb upstream of the ATG site [36]. In our study, rosiglitazone enhanced celecoxib-induced up-regulation of PTEN in N1-S1 cells, and GW9662 attenuated celecoxib-induced up-regulation of PTEN. This indicates that celecoxib can up-regulate PTEN mediated activation and up-regulation of PPARγ in hepatoma cells. Liver specific PTEN deletion promotes CD133^+^ hCSCs in mice [37], and knockdown of PTEN can up-regulate CD133 expression in hepatoma cells [23]. We also found Ad-PTEN gene delivery suppressed hCSCs marker expression and tumor initiating, and PTEN knockdown up-regulated CD133 and CD44 in hepatoma cells. In human Huh-7 cells, Ad-PTEN gene delivery suppressed PGE_2_-induced Akt activation, CD133/CD44 up-regulation and tumor sphere formation. PTEN siRNA transfection partially prevented the effect of celecoxib on sphere formation in N1-S1 and Huh7 cells. This indicates celecoxib-induced PTEN up-regulation is partially involved in the suppression of hepatic cancer stemness. PTEN is not only upstream regulator of Akt, recent studies indicated other molecular such as Aurora A also regulate Akt pathway [38]. mTOR (a downstream effector of Akt) is also highly activated in aggressive HCC, and mTOR activation plays an important role in hepatoma cell growth and development [39]. We also found rapamycin (mTOR inhibitor) suppressed tumor sphere in Huh7 cells. Moreover, PTEN/Akt signaling is also involved in CD44^+^/CD133^+^ CSCs in other solid tumors, not only in liver cancer [40]. Rapamycin also suppressed sphere formation in other cancer [41].

In chemoprevention studies, a 17-day celecoxib treatment potently suppressed tumor burden and tumor weight in rats with Novikoff hepatoma. This is consistent with a previous study showing that diethylnitrosamine induced HCC [18]. Additionally, a preventive celecoxib regimen inhibits CD44 and CD133 expression. Therapeutic celecoxib can retard tumor growth and inhibit CD44^+^/CD133^+^ hCSCs. Moreover, CD133^+^ CSCs reportedly differentiate into endothelial cells in tumors [27]. We also found that celecoxib therapy decreased CD31^+^/CD133^+^ blood vessels in tumor tissues, indicating the trans-differentiation potential of hCSCs. Suppression of hCSCs may be involved in the inhibition of cell proliferation and angiogenesis, as well as induction of cell apoptosis *in vivo* following celecoxib therapy. hCSCs and tumor microenvironments interaction is very important in HCC progression [42], and cancer stemness also promotes immunosppressive cells infiltration (tumor-associated macrophages and regulatory T cells) [43, 44]. Immune-competent orthotopic HCC model can provide a truest stemness niches and tumor microenvironments, and it is an advantage in stem cells biology and tumor immunology studies. In this study, the dose and duration of celecoxib therapy required to suppress cancer stemness is clinically feasible because the pharmacokinetic of celecoxib indicates that the serum celecoxib levels can reach up to 10 μM in rats after feeding at 30 mg/kg [45]. Moreover, it has been recently reported that a 3-day treatment was sufficient to downregulate CD133 expression in patients with colon cancer [46]. This again supports that the dosage of celecoxib used in our study is in the range of physiological concentration, and celecoxib treatment for 7 days is sufficient for suppressing an increase in hCSCs during the short-term therapy. Given the lack of hepatotoxicity and bone marrow suppression, celecoxib therapy may constitute an adjuvant therapy for current HCC therapeutic modalities. Although celecoxib therapy fails to eradicate established HCC, further investigation examining the potential of celecoxib as adjuvant therapy for current HCC therapies such as surgery, TAE, target therapy or chemotherapy is expected, particularly because celecoxib is well tolerated in humans and shows an excellent safety record and limited hepatic toxicity [47]. MK-2206 (a Akt inhibitor) has been reported that it can synergize with convention chemotherapy in HCC [48]. Celecoxib inhibited Akt activation in this study, celecoxib and chemotherapeutic drug combined therapy is maybe a potential treatment for HCC in future. Liver fibrosis/cirrhosis is often observed in HCC patients, celecoxib also shows antifibrogenic effects in rats [49, 50], implicating celecoxib should be considered to treat HCC or other liver diseases in future.

We herewith proposed a model for celecoxib-mediated inhibition of hCSC expansion in Novikoff hepatoma (Fig. 7C). In the extrinsic pathway, celecoxib reduces PGE_2_ production by inhibiting the activities of COX-2 in inflammatory non-tumor tissues. In the intrinsic pathways, celecoxib activates the PPARγ/PTEN pathway, leading to perturbation of the PGE_2_-stimulated Akt signaling. Because of such a dual function of celecoxib in modulating cancer stemness, the therapeutic efficacy of celecoxib for rat Novikoff hepatoma. Because of its similar potency in repressing cancer stemness in human HCC, our study advocates that celecoxib may be constitute an alternative therapeutic agent in human HCC.

In conclusion, we found that celecoxib therapy inhibits hCSCs expansion through both extrinsic and intrinsic pathways; we identified a novel pathway for Akt-signaling regulation by PPARγ/PTEN, which involved, in part, the intrinsic pathway regulating hCSCs. This is the first study to evaluate the therapeutic efficacy and survival analysis of celecoxib in orthotopic HCC. Celecoxib shows a high potential for use in a monotherapy or adjuvant therapy for treating HCC. Because limited therapeutic options are available for treating HCC, celecoxib or COX-2 inhibitors may present a promising alternative for treating HCC. Future clinical studies are necessary to validate the therapeutic potential of celecoxib for HCC and other aggressive cancers.

## MATERIALS AND METHODS

### Cell culture and drugs

N1-S1 (rat HCC), Clone-9 (rat hepatocyte), Hep3B (human HCC) and Huh7 (human HCC) cells were from American Type Culture Collection (ATCC) where they were characterized by mycoplasma detection, DNA Fingerprinting, isozyme detection and cell vitality detection. All cell lines were immediately expanded and frozen such that they could be restarted every 2 to 3 months from a frozen vial of the same batch of cells. N1-S1 cells were maintained in RPMI-1640 medium (Gibco, Bethesda, MD) containing 10% calf serum (Hyclone). Clone-9 cells were maintained in F12K medium (Gibco, Bethesda, MD) containing 10% fetal bovine serum (Hyclone). Hep3B and Huh7 cells were maintained in DMEM medium (Gibco, Bethesda, MD) containing 10% calf serum. All the media for cell culture were supplemented with 2 mM l-glutamine (Hyclone), 100 mg/mL streptomycin (Hyclone), and 100 U/mL penicillin (Hyclone). All cells were maintained under humidified conditions in 95% air and 5% CO_2_ at 37°C. Celecoxib powder was from Pharmacia Corp (St. Louis, MO). Celecoxib capsules (200 mg tablets; Celebrex) were purchased from Pfizer (La Jolla, CA). GW9662, rosiglitazone, verapamil, and rapamycin were purchased from Sigma (St. Louis, MO). PGE_2_ was purchased from Merck (Darmstadt, Germany).

### Cell proliferation Assay

To access the growth rates, cells (5×10^3^/well) were seeded in 96-well plates, and then cells were incubated overnight in 95% air and 5% CO_2_ at 37°C before drug treatment. After drug treatment for 48 hours, Alarmar Blue reagent (10:1) (Invitrogen) was added and cells were incubated at 37°C for 2 hours. Absorbance was measured with an ELISA reader (Dynex Technologies, Inc., Chantilly, VA) at 570-600 nm. Cell viability was expressed as a percentage of absorbance in treated wells relative to that of untreated (control) wells.

### Colony formation assay

Hep3B and Huh7 cells (3000 cells per well) were treated with celecoxib for 10 days, and cells were stained with crystal violet and aggregates of more than 50 cells were scored as colonies.

### Real-time quantitative RT-PCR

Total RNA was extracted from cultured cells using Trizol (Tel-Test Inc., Friendswoods, TX). Two micrograms of total RNA was used for the reverse transcription reaction with Superscriptase II (Invitrogen, Carlsbad, CA) using oligo-dT and random primers. One twentieth of complementary DNA generated was used as template for real time PCR analysis. Amplification and detection were done by a LightCycler DNA Master SYBR Green I kit (Roche, Manheim, Germany) in a LightCycler Detection System (Roche, Manheim, Germany). The PCR reaction was carried out as follows: one cycle of 95 °C for 10 min, 45 cycles of 95°C for 15 s, 60 °C for 5 s, and 72 °C for 20 s. After amplification, a final melting curve protocol was performed to determine the specificity of the PCR reaction. The primer sequences were as follows: β-actin, (forward primer: 5′-TCCTGTGGCATCCACGAAACT-3′; reverse primer: 5′-GAAGCATTTGCGGTGGACGAT-3′); COX-2, (forward primer: 5′-GGTGTATCCCCCCACAGTCA-3′; reverse primer: 5′-CCAGG CACCAGACCAAAGAC-3′).

### PGE2 quantification

Measurement of secreted PGE_2_ in culture supernatant was done by competitive enzyme immunoassay (EIA) kit (Cayman Chemical, Ann Arbor, MI).

### Sphere formation assay

N1-S1 or Huh7 cells were suspended in serum-free stem cell medium containing DMEM/F12 supplemented with N2 supplement (Gibco), EGF (20 ng/mL; PeproTech), and bFGF (20 ng/mL; PeproTech). To study the effect of celecoxib or rapamycin on PGE_2_-promoted sphere formation, cells were pretreated with PGE_2_ (1 μM) in serum-free medium for 48h after serum starvation for 24 h. After PGE_2_ pretreatment, cells (5000 cells per well) were seeded into an ultralow-attachment 6-well plate (Corning Life Sciences, Lowell, MA) with or without celecoxib (10 μM) or rapamycin (100 nM) treatment. After 7 to 10 days, the number of tumor spheres was evaluated using light microscopy (Leica Microsystems). When cells were infected with adenovirus (Ad-GFP or Ad-PTEN), PGE_2_ (1 μM) was added at 12 h post infection, and cells were treated in serum-free medium for 48 h before sphere culture. When cells were transfected with siRNA, cells were seeded into an ultralow-attachment 6-well plate with or without celecoxib (10 μM) at 12 h post transfection.

### Side population cells (SPCs) analysis

After treatment with celecoxib (10 μM) or PGE_2_ (1 μM) in serum-free medium for 48 hours, cells (1 × 10^6^ cells/mL) were incubated in DMEM/5% FBS containing fresh Hoechst 33342 (final concentration 5 μg/mL; Sigma-Aldrich) for 90 min at 37 °C. In some cases, cells were incubated with the Hoechst dye in the presence of verapamil (50 μM; Sigma-Aldrich) for reliable gating of SPCs. After indicated treatment, cells were centrifuged and resuspended in PBS containing propidium iodide (1 μg/mL; Sigma-Aldrich) for 5 min before flow cytometric analysis (Becton Dickinson). The Hoechst dye was excited with the UV laser at 351–364 nm, and fluorescence was measured using a 515-nm side population filter (Hoechst blue) and a 608 EFLP optical filter (Hoechst red). A 540 DSP filter was used to separate the emission wavelengths. Data were collected and analyzed using the CellQuest software (Becton Dickinson).

### Retrovirus production and infection

Retrovirus vectors encoding CD133 was generated by co-transfection of pCX-puro-CD133 with pVSV-G (envelope) and pVSV-GP (packaging) plasmids in 293T cells using lipfectamine 2000 (Invitrogen). N1-S1 cells were infected with virus-containing medium for 24 hours and stable expression clones were selected with 2 μg/ml puromycin (Invitrogen) for 7 days.

### Immunoblot analysis

Protein was isolated using buffer containing 150 mM NaCl, 50 mM HEPES, pH 7, 1% Triton X-100, 10% glycerol, 1.5 mM MgCl_2_, 1 mM EGTA, and protease inhibitors (Roche, Mannheim, Germany). After the proteins were separated using 8%- 10% SDS-PAGE, they were transferred onto polyvinylidene fluoride membranes by using a blotting apparatus. The membrane was blocked with 5% milk in Tris-buffered saline/Tween-20 for 1 h and then incubated with a 1:500 dilution of CD44, CD133, Akt, ALDH, VEGF, PPARγ, HA-tag or GFP antibody (all from Santa Cruz Biotechnology); a 1:1000 dilution of pAkt (ser473), PTEN, or PCNA antibody (all from Cell Signaling, Beverly, MA); a 1:5000 dilution of β-actin antibody (Sigma-Aldrich) for 1 h at room temperature or overnight at 4°C. After the secondary antibody was conjugated with HRP (1:5000 dilution in 5% milk; Santa Cruz Biotechnology) for 60 min, the signals on the membrane were detected using ECL-Plus luminol solution (GE Healthcare, Little Chalfont, Buckinghamshire, UK) and exposed to X-ray film for autoradiography. Quantification was performed using the Quantity-One software (Bio-Rad Laboratories, Hercules, CA).

### Animal experiments

All experimental procedures were reviewed and approved by the Institutional Animal Care and Use Committee before the study began, and we ensured that all animals received humane care and that study protocols complied with the institution's guidelines. The US-guided induction of Novikoff hepatoma in Sprague Dawley (SD) rats was performed as previously described [21]. In chemopreventive regimen, rats (n =12) were implanted with N1-S1 cells on day 0, then divided into two groups receiving saline (n = 6) or celecoxib (30 mg/kg/day; n = 6) via oral route for 17 days, and sacrificed on day 18 for histological analysis. For therapeutic studies, rats (n = 32) were implanted with N1-S1 cells on day 0. After confirming HCC formation by US monitoring on day 10, rats (n = 26) were divided into two groups receiving saline (n = 13) or celecoxib (30 mg/kg/day; n = 13) via oral route for additional 7 days, and sacrificed on day 18 for histological analysis. For survival analysis, rats (n = 24) were used for induction of Novikoff hepatoma on day 0. After confirming HCC formation by US monitoring on day 10, rats (n = 18) were divided into two groups receiving saline (n = 9) or celecoxib (30 mg/kg/day; n = 9) via oral route for additional 60 days. In adenovirus gene delivery study, N1-S1 cells were infected with adenovirus vectors at a multiplicity of infection (MOI) of 200 for 24 h, implanted into rat under US guidance on day 0. The hepatoma induction was determined after day 10.

### Histological analysis

For immunohistochemical analysis, paraffin-embedded hepatoma tissue blocks were sectioned into 3-μm-thick slices and mounted on poly(lysine)-coated slides. After the slides were deparaffinized, they were blocked with 3% hydrogen peroxide for 10 min and subjected to antigen retrieval in 10 mM citrate buffer for 15 min in a microwave. The slides were incubated with a 1:50 dilution of CD31, CD133 or CD44 antibody (all from Santa Cruz Biotechnology, Santa Cruz, CA); or a 1:50 dilution of Ki-67 antibody (Cayman Chemical, Ann Arbor, MI) at 4°C overnight. After the sections were washed with PBS, they were incubated with horseradish peroxidase/Fab polymer conjugate (polymer detection system; Zymed Laboratories, South San Francisco, USA) for 30 min and detected using diaminobenzidine (1:20 dilution; Zymed Laboratories, South San Francisco, USA). For immunofluorescence detection of CD31, CD133, and CD44 in a paraffin-embedded hepatoma section of HCC, protein blocking was performed for 30 min in 10% fetal calf serum after antigen retrieval in 10 mM citrate buffer for 15 min in a microwave. CD31, CD133, and CD44 antibodies (1:50 dilution for all; Santa Cruz Biotechnology) were applied to the sections, which were then incubated at room temperature for 2 h, followed by repeated washing with phosphate-buffered saline (PBS). Next, tissue sections were incubated with Alexa Fluor 546- or Alexa Fluor 488-conjugated IgG (Molecular Probes, Eugene, OR) for 30 min at room temperature. After the slides were mounted in mounting media (Dako, Glostrup, Denmark), they were visualized under a fluorescence microscope (Leica Microsystems, Schweiz, AG – CH).

### Counting of CD44^+^/CD133^+^ hCSCs by flow cytometry

After 10 μM celecoxib or 1 μM PGE_2_ treatment in serum-free medium for 48 hours, cells were incubated with CD44 and CD133 (1:100 dilution for both; both were from Santa Cruz Biotechnology) antibodies for 30 min at room temperature and then washed 3 times with PBS. Next, signals were detected using an Alexa Fluor 488- or 546-conjugated IgG (Molecular Probes). Then, we used FACS Calibur flow cytometry (Becton Dickinson, San Jose, CA) to determine the ratio of CD44^+^/CD133^+^ hCSCs. All the data were collected and analyzed using the CellQuest software (Becton Dickinson).

### Cell immunofluorescence analysis

After 10 μM celecoxib or vehicle treatment for 48 hours, cells were fixed in 4% paraformaldehyde for 10 min and stained with PPARγ, and PTEN antibodies (1:50 dilution for all; all the antibodies were from Santa Cruz Biotechnology), followed by an Alexa Fluor 488 or 546-conjugated IgG (Molecular Probes). The cells were counterstained with DAPI and visualized using a confocal microscope (Carl Zeiss, Jena, Germany). Quantification was performed using the ImageJ software (NIH).

### Terminal deoxynucleotidyl transferase-mediated dUTP nick end-labeling (TUNEL) staining

Rat hepatoma sections were placed on slides, deparaffinized, and washed with PBS. TUNEL analysis was performed using the *in situ* Cell Death Detection Kit, Fluorescein (Roche, Indianapolis, IN), according to the manufacturer's protocol. TUNEL-positive cells were visualized using immunofluorescent microscopy and counted using a 20× objective. TUNEL-positive cells containing FITC were identified by colocalization with 4,6-diamidino-2-phenylindole (DAPI) staining and on the basis of morphological features. More than 100 cells were counted for each variable per experiment. The slides were viewed under a fluorescence microscope (Leica Microsystems), with green fluorescence set at 520 nm. The cells that were stained green indicated apoptotic cells. For comparison, the amount of positive staining was counted under low power fields.

### Adenovirus production and infection

Production and infection of adenovirus vectors were performed as previously described [51].

### Transfection with siRNA

N1-S1 cells (in 6-well plate) were transfected with scramble siRNA and PTEN siRNA (Santa Cruz Biotechnology, Santa Cruz, CA) for 72 hours using the lipofectamine 2000 (Invitrogen, Carlsbad, CA) according to the manufacturer's instructions.

### Statistical Analysis

Differences between the groups were statistically evaluated using the unpaired Student's *t* test. Survival analysis was performed using the Kaplan–Meier method in SPSS V.19 software. Data were mean ± SD. All *P* values were two-tailed (*p < 0.05, **p < 0.01).

## SUPPLEMENTARY FIGURES


