# Supplementary material for: Celecoxib suppresses hepatoma stemness and progression by up-regulating PTEN
Source: Oncotarget. 2013 Dec 28;5(6):1475–90. doi: 10.18632/oncotarget.1745 (PMC4039225; doi:10.18632/oncotarget.1745)
Supplement: Supplementary file 1 [file oncotarget-05-1475-s001.pdf]

Celecoxib suppresses hepatoma stemness and progression by up-regulating PTEN – Chu et al

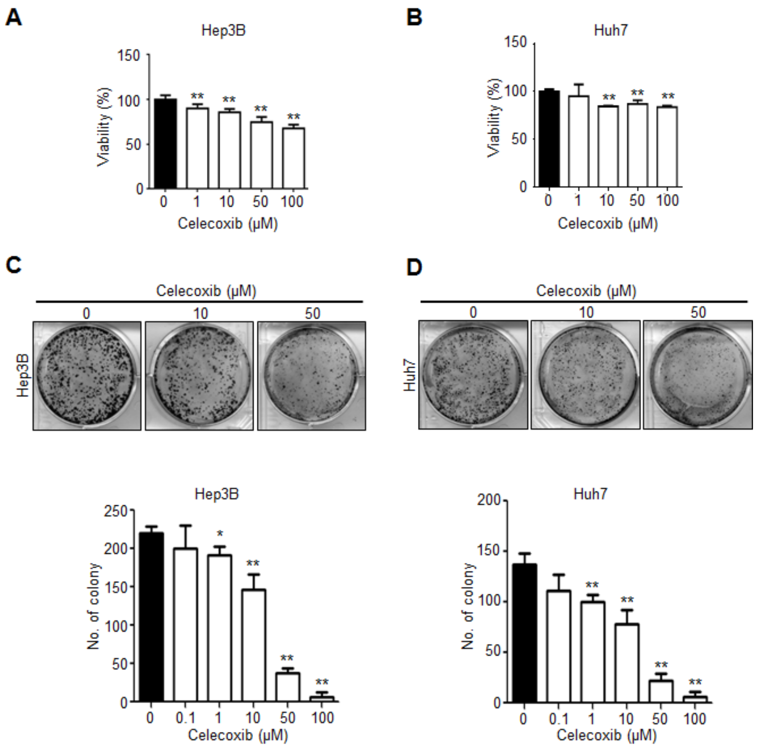

Supplementary Fig. 1: Celecoxib inhibited the proliferation and anchorage-independent cell growth in hepatoma cells. (A, B) The proliferation of human HCC cells after celecoxib treatment for 48 hours was determined by alamar blue assay. (C, D) The anchorage-independent growth of human HCC cells after celecoxib treatment for 7 days was determined by flat colonies formation assay. Data were mean ± SD (*$p < 0.05$, **$p < 0.01$).

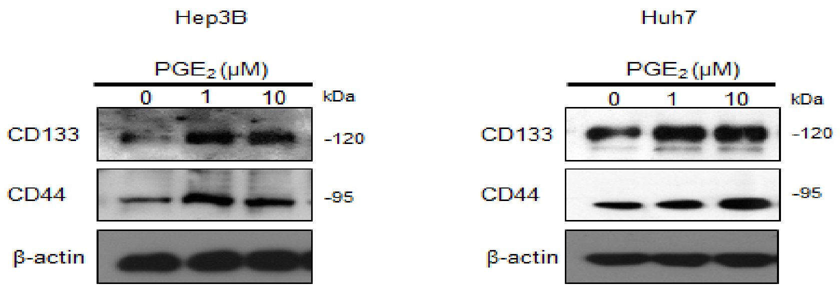

Supplementary Fig. 2: PGE_2 induced CD133 and CD44 up-regulation in human HCC cells. Immunoblot analysis of CD44 and CD133 expression in Hep3B and Huh7 cells treated with different dose of PGE_2 for 48 h.

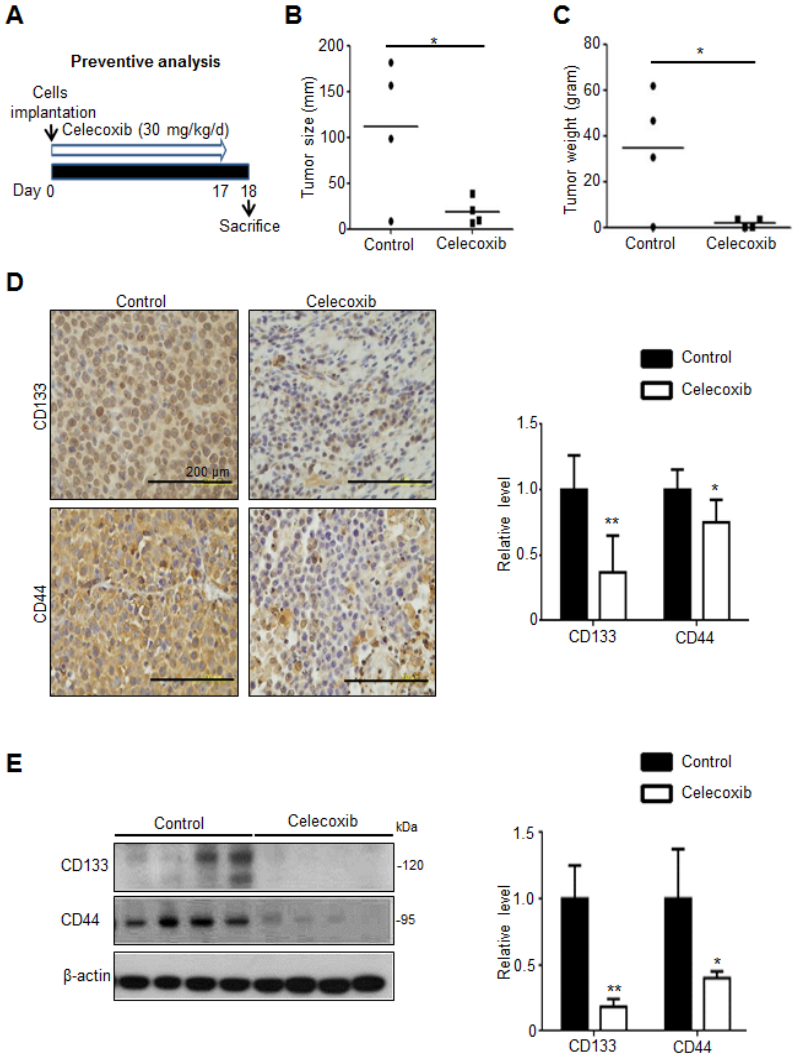

Supplementary Fig. 3: Prophylactic celecoxib therapy reduced tumor burden and inhibited cancer stemness markers CD44/CD133 expression in Novikoff hepatoma. (A) Experimental scheme. (B) tumor size measured by caliper, and (C) tumor weight after animal sacrificing. (D) Immunohistochemestry staining and (E) Western blot analysis of CD133 and CD44 in Novikoff hepatoma tissues after chemoprevention by celecoxib. Data were mean ± SD (*p < 0.05, **p < 0.01).
